# Thermodynamic Modeling and Experimental Validation for Thermal Beneficiation of Tungsten-Bearing Materials

**DOI:** 10.3390/ma18040899

**Published:** 2025-02-19

**Authors:** Ndue Kanari, Frederic Diot, Chloe Korbel, Allen Yushark Fosu, Eric Allain, Sebastien Diliberto, Eric Serris, Loïc Favergeon, Yann Foucaud

**Affiliations:** 1 Université de Lorraine, CNRS, GeoRessources, F-54000 Nancy, France; frederic.diot@univ-lorraine.fr (F.D.); chloe.korbel@univ-lorraine.fr (C.K.); allen.fosu@univ-lorraine.fr (A.Y.F.); ericgallain@gmail.com (E.A.); 2 Université de Lorraine, CNRS, IJL, F-54000 Nancy, France; sebastien.diliberto@univ-lorraine.fr; 3 Mines Saint-Étienne, Univ Lyon, UMR 5307 LGF, Centre SPIN, F-42023 Saint-Étienne, France; serris@emse.fr (E.S.); favergeon@emse.fr (L.F.)

**Keywords:** scheelite, tungsten, critical and strategic materials, thermal beneficiation, magnesium chloride, thermodynamic assessment, extraction

## Abstract

Tungsten (W), a rare metal, is categorized as a Critical and Strategic Raw Material (CRM) by the European Union (EU), with the highest economic importance of all selected CRMs since 2014. Tungsten and its derivatives are extracted from their commercial raw materials, mainly wolframite [(Fe,Mn)WO_4_] and scheelite (CaWO_4_) ores. Subsequently to mining and mineral processing, the W ore is submitted to thermal treatment and hydrometallurgy under aggressive conditions (high pressure and temperature), which are usually applied for the extraction of tungsten compounds. This paper aims to investigate a thermal route for scheelite processing using various selected chemical agents, resulting in a W-bearing material that is capable of being leached under softer conditions. In this context, a thermodynamic study of the interaction between FeWO_4_, MnWO_4_ and CaWO_4_ and various chemical reagents is described. The thermochemical calculations and data modeling show that, among other considerations, the reaction of CaWO_4_ with magnesium chloride (MgCl_2_) can lead to the formation of magnesium tungsten oxide (MgWO_4_), which appears to be more easily leachable than CaWO_4_. Experimental tests of the reaction of scheelite with MgCl_2_ appear to validate the thermodynamic predictions with satisfactory process kinetics at temperatures from 725 to 775 °C.

## 1. Introduction

Energy transition, the manufacturing of smart materials, diverse advanced industries and the development of highly innovative technologies, require increasingly large amounts of several raw materials (mainly metallic raw materials). According to European (EU) assessments [1], a good number of these raw materials are considered to be Critical and Strategic Raw Materials (CRMs). Such a designation results from their great economic importance, combined with a high supply risk. The number of CRMs for the EU has grown from 14 CRMs in 2011 to 27 in 2017, and was augmented thereafter to reach 34 CRMs in 2023. Please note that copper and nickel do not meet the critical thresholds, but they are included in CRMs as Strategic Raw Materials.

France, like other EU countries, is strongly dependent on CRMs for its industrialization and support of emerging technologies. Efforts to enable the sustainable supply of these materials from domestic and international sources are currently ongoing. National scientific programs, including the Priority Research Program and Equipments (PEPR), under the management of the French National Research Agency, ANR, launch initiatives to consolidate the country’s scientific and technological base. One of the PEPR programs (The Subsoil (resources) exploratory PEPR), entitled “Innovative and sustainable Technologies platform—InnovTech”, aims to develop new approaches and technology to reduce energy consumption and waste in different stages of metal extraction. The project is coordinated by the BRGM (The French Geological Survey Office) and the co-leader is the GeoRessources laboratory at the “Université de Lorraine” (France). One of the targeted metals of this program is tungsten (W) contained in scheelite skarns from the “French Massif Central” deposit. Please note that tungsten has the highest economic importance among all of the selected CRMs.

Tungsten (meaning heavy stone in Swedish) is also known as wolfram (from wolframite, said to be named from *wolf rahm* or *spumi lupi*, because the ore interfered with the smelting of tin and was supposed to devour the tin) [2]. Tungsten has the highest melting point “m.p.” (3422 °C) of all metals, boils around 5555 °C and is a heavy metal with a specific gravity of 19.3. Tungsten is one of the transition and refractory metals and belongs, along with chromium and molybdenum, to group 6 on the periodic table. All of these elements are known to have diverse valences, which, in common compounds, range mainly from +2 to +6.

Tungsten belongs to the rare elements, with an earth’s crust abundance of 1.25 mg/kg, but it is widespread within all continents. Tungsten occurs in wolframite [(Fe,Mn)WO_4_], scheelite (CaWO_4_), hubnerite (MnWO_4_) and ferberite (FeWO_4_) [2].

**Figure 1:**
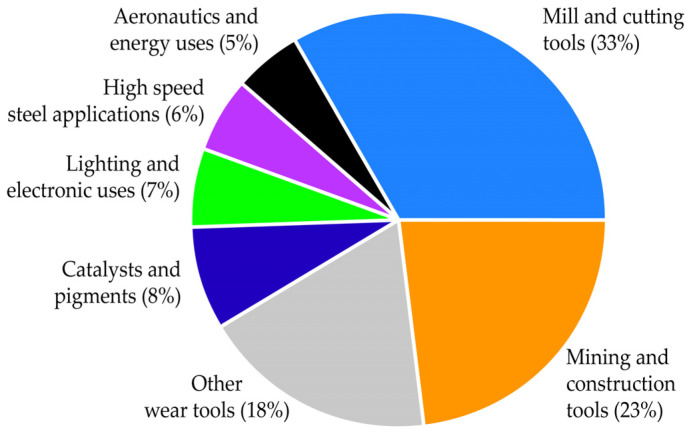
Tungsten-use in various applications in the European Union.

As described in Reference [3], the amount of tungsten consumed in the EU is used for a wide range of applications, which are as follows: 67% of tungsten is used for the manufacturing of tungsten carbides, 11% is used for the manufacturing of W-metal, 11% is used for the manufacturing of chemicals, 8% is used for the manufacturing of tungsten-based steel applications and the rest (3% of W) is used for the manufacturing of other tungsten alloys. The repartition of tungsten-use in the EU in various applications is shown in Figure 1 [3]. Mill and cutting tools, mining and construction and wear tools, with 33%, 23% and 18%, respectively, share the main applications of tungsten-bearing materials. The remaining material, about a quarter of tungsten’s end uses, is distributed across other applications, as shown in Figure 1.

**Figure 2:**
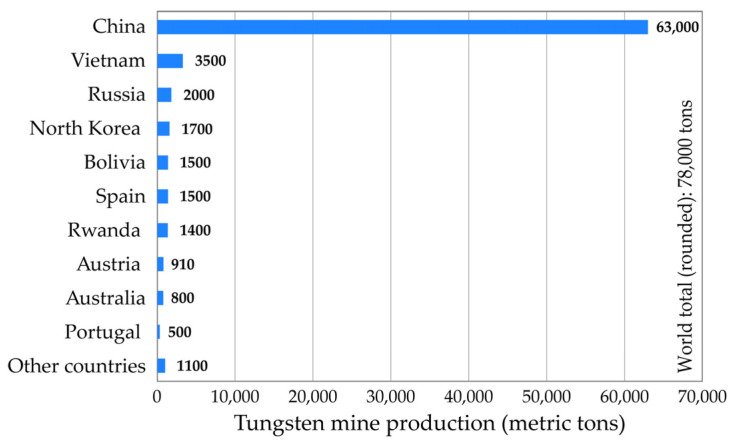
World mine production of tungsten in 2023.

According to the available data [4], the world mine production of tungsten is estimated at 78,000 tons in 2023 (Figure 2). The mine production of tungsten is dominated by China, providing more than 80% of the global W-production. As far as the European Union is concerned, the main mining production of tungsten is concentrated mostly in Austria, Portugal and Spain.

**Figure 3:**
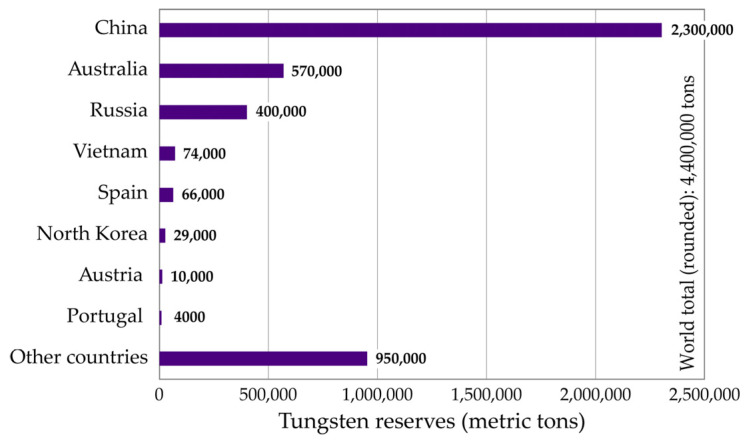
World reserves of tungsten.

Although tungsten resources and reserves are scattered across many countries, China remains prevalent [4], with nearly 52% of the world’s reserves, estimated at 4.4 × 10^6^·tons (Figure 3). Australia and Russia hold second and third place, with their W-reserves estimated at around 13% and 9%, respectively.

As mentioned above, the main tungsten ores of industrial importance are scheelite (CaWO_4_) and wolframite [(Fe,Mn)WO_4_), the latter being a solid solution of the isomorphic minerals, namely ferberite and hubnerite. Scheelite and wolframite account for 70% and 30% of the world resources, respectively. In general, most deposits have low WO_3_ content, which can, at most, reach 2% WO_3_ for richer W-ores; however, the extractive metallurgy of tungsten requires concentrates with around 65% WO_3_ content.

Therefore, the ores must first go through physical enrichment processes that are often complicated due to their complex elemental, chemical and mineralogical compositions [5]. All of the known mineral processing methods, as well as the development of advanced flow-sheets for the treatment of scheelite ores, resulting in a W-concentrate of metallurgical grade, were summarized in Reference [5].

**Figure 4:**
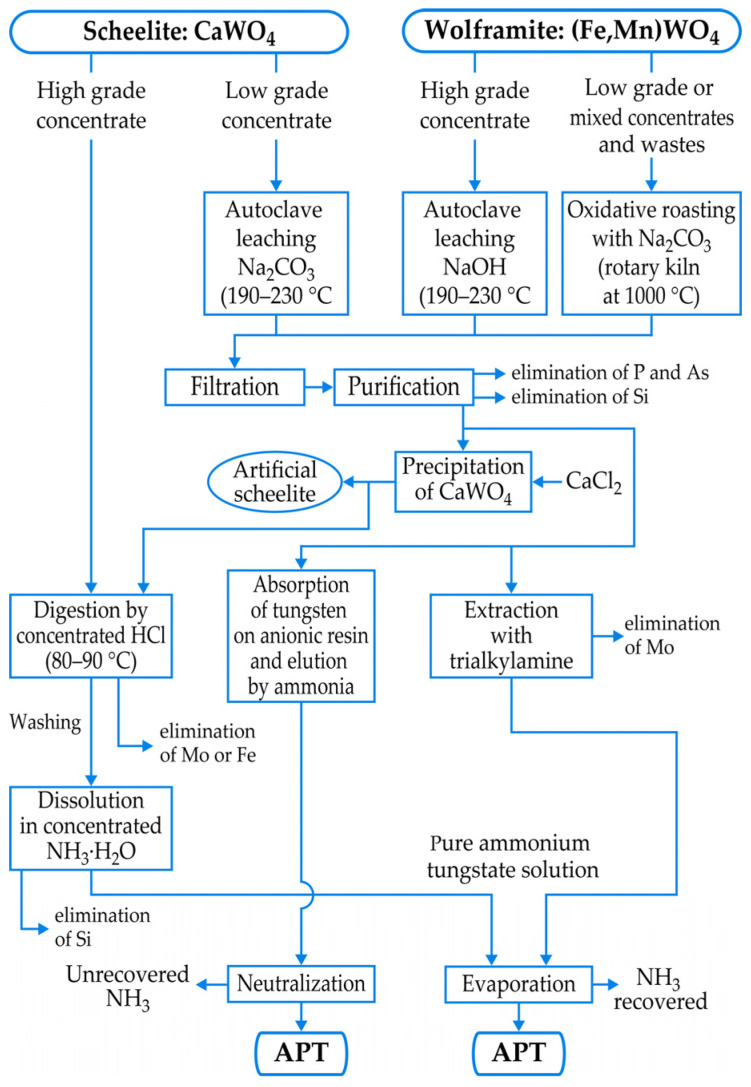
Treatments of tungsten concentrates.

However, the real challenge of the tungsten industry is the metallurgical extraction of tungsten intermediates, such as ammonium paratungstate (APT), with a chemical formula of (NH_4_)_10_[H_2_W_12_O_42_]·4H_2_O, being the most important precursor for the majority of tungsten products. Figure 4 [6] groups simplified schemes for treatments of low- and high-grade scheelite and wolframite concentrates, resulting in APT manufacturing. In general, concentrates undergo a pretreatment (thermal and/or acidic) to remove impurities (S, As, P, organic carbon, etc.) that would be difficult and expensive to remove later. The hydrochloric acid digestion around 90 °C is industrially employed, mainly for the high-grade scheelite concentrates [6,7], producing pure tungstic acid (H_2_WO_4_ or WO_3_·H_2_O) and/or APT after dissolving H_2_WO_4_ into concentrated NH_3_·H_2_O.

The most current commercial process by soda/caustic soda digestion (high-pressure and high-temperature processes mainly applied), followed by solvent extraction or an ion exchange step, can accommodate a variability of raw materials. However, high reagent consumption, the absence of recycling the aqueous solutions and chemical reagents, as well as discharge of high-salinity wastewater for solvent extraction and ion exchange steps, are some of the process drawbacks [7]. Novel laboratory routes for improving the existing processes, leading to better leaching and efficiency recovery of tungsten from its concentrates, are well-documented in Reference [7].

There are also some thermochemical insights and experimental laboratory works describing new thermal alternative methods to replace the current industrial processes for tungsten extraction from its bearing materials. An early study [8] predicted the reactions of gangue minerals (SiO_2_, Ca_5_(PO_4_)_3_F, SnO_2_, FeS_2_, MoS_2_ and FeAsS) with CaCO_3_ and Ca_3_WO_6_, resulting in mixed compounds which nature depends on for the oxygen partial pressure in the selected system. Xu et al. [9] performed a thermodynamic analysis of leaching of the thermally synthetized Ca_3_WO_6_ in NH_3_-(NH_4_)_2_CO_3_-H_2_O solution. Their experimental results, in which they simultaneously applied grinding and leaching, indicated an almost full dissolution of Ca_3_WO_6_ at a temperature close to 40 °C by forming (NH_4_)_2_WO_4(aq)_ and solid CaCO_3_. Another scientific approach [10] provided a leaching yield of MgWO_4_ that was higher than 98% in a solution of NaOH at 90 °C, after thermal synthesis of magnesium tungstate from scheelite using MgCl_2_.

Recently [11], a carbo-thermic reduction in the presence of calcium sulfate was applied to a wolframite–scheelite mixed ore containing tin as cassiterite (SnO_2_). About 99% of tin is separated by volatilization as SnS. Wolframite is converted to CaWO_4_ and Ca_2_(Fe,Mn)WO_6_, which are easily digested by the mixed sulphuric–phosphoric acid solution with a leaching efficiency of tungsten of around 99%. The kinetics of chlorination of a scheelite–wolframite concentrate with chlorine and sulphur dioxide are investigated early [12]. They identified WO_2_Cl_2_, FeCl_2_, FeCl_3_, S_2_, FeS, CaSO_4_ and CaCl_2_ as the main reaction products. In a similar study, Menéndez et al. [13] performed the physicochemical characterization and chlorination of several natural wolframite (Mn_x_Fe_1−x_WO_4_) samples with chlorine and sulphur dioxide. Under the best conditions, they announced an extraction extent of tungsten that was close to 86%. Fuga et al. [14] studied the chlorination kinetics of a MnWO_4_ reaction with chlorine between 650 and 950 °C, and they identified WO_2_Cl_2_ and MnCl_2_ as the main reaction products.

The aim of this paper is to provide a comprehensive thermochemical assessment of the interaction between tungsten-bearing minerals and diverse chemical reagents. After predicting the thermodynamic reactivity of tungsten compounds towards selected chemical agents, special attention was paid to the modeling of the equilibrium composition of various tungsten-containing systems at chosen temperatures. Furthermore, experimental tests of the reactions of scheelite with calcium carbonate (CaCO_3_) and calcium chloride (MgCl_2_) are carried out to verify the validity of the thermodynamic predictions.

## 2. Phase Diagrams of the CaO-WO_3_ and MgO-WO_3_ Systems

Both calcium and magnesium oxides and their mixture are largely used in the manufacturing of basic refractory bricks due to their chemical and mechanical performance, as well as their high melting points (2613 °C for CaO and 2852 °C for MgO). In addition, the ternary and quaternary phase diagram systems, CaO-MgO-SiO_2_, CaO-MgO-SiO_2_-Al_2_O_3_ and many others, are the basis of extractive metallurgy (especially in slag formation) and in the synthesis of glass and glass–ceramic materials.

**Figure 5:**
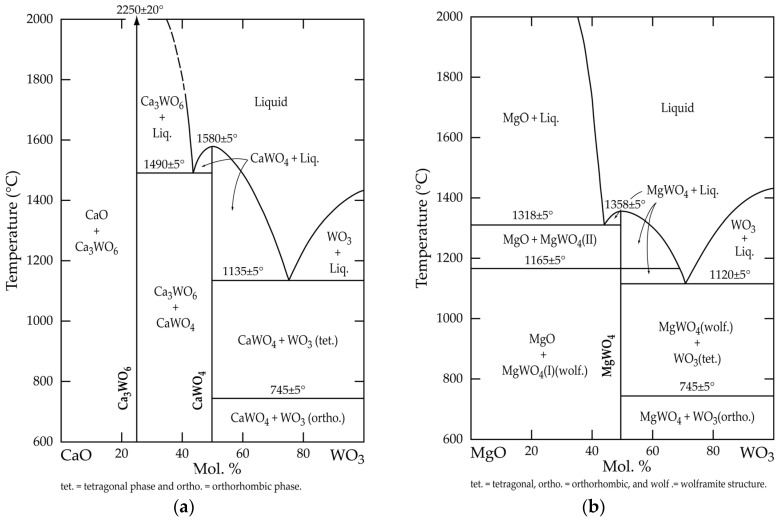
Phase diagram of (**a**) CaO-WO_3_; (**b**) MgO-WO_3_ system (adapted from Reference [15]).

In the context of this study, it is interesting to examine the available binary phase diagrams of CaO(MgO)-WO_3_ systems. In the CaO-WO_3_ phase diagram depicted in Figure 5a, there are two well-defined and stable compounds, CaWO_4_ and Ca_3_WO_6_ (3CaO·WO_3_), which melt congruently at about 1580 °C and 2250 °C, respectively. According to this diagram, both compounds do not show any crystalline structural change, whilst the tungsten (VI) oxide (WO_3_) crystal structure is temperature-dependent. Furthermore, it is orthorhombic at 600–745 °C and it is transformed into a tetrahedral state beyond this temperature.

The MgO-WO_3_ phase diagram presented in Figure 5b demonstrates the formation of a single compound, namely magnesium tungstate (MgWO_4_), which is stable up to its melting point (ca. 1385 °C); however, it undergoes a reversible structural phase transition at around 1165 °C. The features of this transformation are described elsewhere [16].

These observations prove that, in addition to scheelite, two other compounds that are thermally stable, at least at T < 1350 °C, must be taken into account in the studied systems. Hence, this statement will be used for the thermodynamic investigation of the interaction between scheelite and the selected chemical reagents.

## 3. Thermodynamic Elements of the Interaction Between (Fe, Mn, Ca) Tungstate and Various Chemical Reagents

As mentioned in the Section 1, one of the aims of this study is to thermally transform tungsten-bearing materials (FeWO_4_, MnWO_4_ and CaWO_4_) into less-common compounds (such as Ca_3_WO_6_ and MgWO_4_) that are more likely to be digested by current chemical reagents under mild conditions. HSC Chemistry Software^®^ (version 5.1) [17], as well other available data [18], are used for the thermodynamic predictions and assessment of the selected system. Although there are other types of computer software and databases for thermodynamic predictions (such as FactSage, Thermo-Calc Software, etc.), HSC Chemistry appears to be the most widely used. The used methodology, as well as the precautions taken into account during the thermochemical calculations, were mentioned in a recent study [19]. The thermodynamic data (enthalpy (H), entropy (S) and heat capacity ©) of some chemical species are sometimes given in a low and narrow range of temperatures, and HSC data extrapolation at high temperatures may lead to errors. As an example linked to this study showed, the thermodynamic data for CaWO_4_ and Ca_3_WO_6_ are available up to 1000 K (727 °C). However, as shown in Figure 5a, these well-defined compounds are thermally stable at temperatures well above 727 °C and do not undergo any structural and/or transition changes prior to melting. Hence, the thermochemical data for both compounds can be extrapolated without error in the temperature range of interest of this investigation (lower than or equal to 1100 °C).

**Figure 6:**
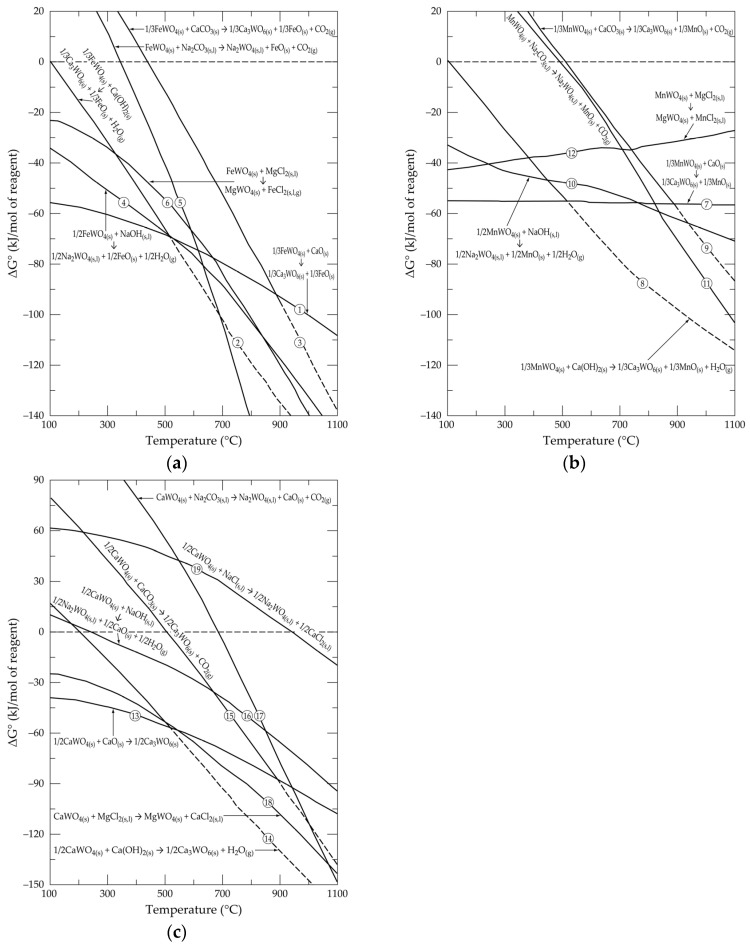
Evolution of standard free energy changes as a function of temperature for the reactions of selected chemical reagents with (**a**) FeWO_4_; (**b**) MnWO_4_; (**c**) CaWO_4_.

The first step for an envisaged process is to check the thermodynamic reactivity of input chemicals by evaluating the standard free Gibbs energy changes (ΔG°), computed by the HSC thermochemical database, for foreseen chemical reactions. Figure 6a–c plots the evolution of ΔG° versus temperature for the reactions of FeWO_4_, MnWO_4_ and CaWO_4_ with selected chemical reagents, as described in Equations (1)–(19). There can certainly also be intermediate reactions, such as the formation of FeCO_3_, MnCO_3_, Fe(HO)_2_ and Mn(OH)_2_, but in the practical temperature of interest (most likely higher than 600 °C), these compounds are dissociated in respective oxides (FeO and MnO, in absence of oxygen) and their respective gases (CO_2(g)_ and H_2_O_(g)_). Likewise, other possible reactions should lead to the formation of more complex intermediate phases, but the thermodynamic data are often lacking. However, as the ΔG° is a state function, the final ΔG° value does not depend on the intermediate paths of the system transformation, but only on the initial and final state of the system.

1/3FeWO_4(s)_ + CaO_(s)_ → 1/3Ca_3_WO_6(s)_ + 1/3FeO_(s)_ (1)

1/3FeWO_4(s)_ + Ca(OH)_2(s)_ → 1/3Ca_3_WO_6(s)_ + 1/3FeO_(s)_ + H_2_O_(g)_ (2)

1/3FeWO_4(s)_ + CaCO_3(s)_ → 1/3Ca_3_WO_6(s)_ + 1/3FeO_(s)_ + CO_2(g)_ (3)

1/2FeWO_4(s)_ + NaOH_(s,l)_ → 1/2Na_2_WO_4(s,l)_ + 1/2FeO_(s)_ + 1/2H_2_O_(g)_ (4)

FeWO_4(s)_ + Na_2_CO_3(s,l)_ → Na_2_WO_4(s,l)_ + FeO_(s)_ + CO_2(g)_ (5)

FeWO_4(s)_ + MgCl_2(s,l)_ → MgWO_4(s)_ + FeCl_2(s,l,g)_ (6)

As shown in Figure 6a, the thermodynamic reactivity of FeWO_4_, with respect to CaO, Ca(OH)_2_, NaOH and MgCl_2_, seems to be high (negative values of ΔG°) from around 100 °C. At a higher temperature (T > 500 °C), all of the considered forward reactions (Equations (1)–(6)) are spontaneous. Similarly, the reactions of MnWO_4_ (Equations (7)–(12)) with the selected reagents occur spontaneously, starting at least from temperatures higher than 500 °C (Figure 6b).

1/3MnWO_4(s)_ + CaO_(s)_ → 1/3Ca_3_WO_6(s)_ + 1/3MnO_(s)_ (7)

1/3MnWO_4(s)_ + Ca(OH)_2(s)_ → 1/3Ca_3_WO_6(s)_ + 1/3MnO_(s)_ + H_2_O_(g)_ (8)

1/3MnWO_4(s)_ + CaCO_3(s)_ → 1/3Ca_3_WO_6(s)_ + 1/3MnO_(s)_ + CO_2(g)_ (9)

1/2MnWO_4(s)_ + NaOH_(s,l)_ → 1/2Na_2_WO_4(s,l)_ + 1/2MnO_(s)_ + 1/2H_2_O_(g)_ (10)

MnWO_4(s)_ + Na_2_CO_3(s,l)_ → Na_2_WO_4(s,l)_ + MnO_(s)_ + CO_2(g)_ (11)

MnWO_4(s)_ + MgCl_2(s,l)_ → MgWO_4(s)_ + MnCl_2(s,l)_ (12)

1/2CaWO_4(s)_ + CaO_(s)_ → 1/2Ca_3_WO_6(s)_ (13)

1/2CaWO_4(s)_ + Ca(OH)_2(s)_ → 1/2Ca_3_WO_6(s)_ + H_2_O_(g)_ (14)

1/2CaWO_4(s)_ + CaCO_3(s)_ → 1/2Ca_3_WO_6(s)_ + CO_2(g)_ (15)

1/2CaWO_4(s)_ + NaOH_(s,l)_ → 1/2Na_2_WO_4(s,l)_ + 1/2CaO_(s)_ + 1/2H_2_O_(g)_ (16)

CaWO_4(s)_ + Na_2_CO_3(s,l)_ → Na_2_WO_4(s,l)_ + CaO_(s)_ + CO_2(g)_ (17)

CaWO_4(s)_ + MgCl_2(s,l)_ → MgWO_4(s)_ + CaCl_2(s,l)_ (18)

1/2CaWO_4(s)_ + NaCl_(s,l)_ → 1/2Na_2_WO_4(s,l)_ + 1/2CaCl_2(s,l)_ (19)

Only the reactions of CaWO_4_ with CaO and MgCl_2_ (Equations (13) and (18)) are thermodynamically feasible at a low temperature (Figure 6c). For some reactions, such as that with Na_2_CO_3_ (Equation (17)) and NaCl (Equation (19)), it is necessary to exceed 700 and 950 °C, respectively, in order for the reactions to occur in the desired direction.

Processes based on the roasting of wolframite and scheelite with alkali compounds (especially with Na_2_CO_3_) are known in the current tungsten extraction processes [6]. Thus, among the chemical processes based on the reactions mentioned above, attempts have been made for those based on the treatment with calcite (CaCO_3_) and magnesium chloride (MgCl_2_). It is therefore interesting to follow the chemical equilibria between the tungsten compounds and these chosen chemical reagents.

## 4. Calculation of the Equilibrium Composition in the (Fe,Mn,Ca)-W-C-O Systems

The HSC software® 5.1 also offers the facility to calculate the equilibrium composition of a multicomponent system. The equilibrium composition is calculated using the GIBBS-solver, which uses the Gibbs energy minimization method [17]. Thus, a chemical system seeks the minimum change in Gibbs free energy (ΔG = 0) at the equilibrium state. For a simple system, the equilibrium constant approach is used to find the equilibrium compositions; however, in complex systems with a large number of constituents distributed in different phases (solid–liquid–gas), the calculation of the equilibrium composition is a difficult one. The GIBBS solver module of the HSC allows us to calculate the equilibrium composition as a function of the amount of species introduced at the chosen temperature and pressure. Although there are many options for the result presentation, the obtained data are often plotted as an evolution of the equilibrium composition and as a function of temperature.

**Figure 7:**
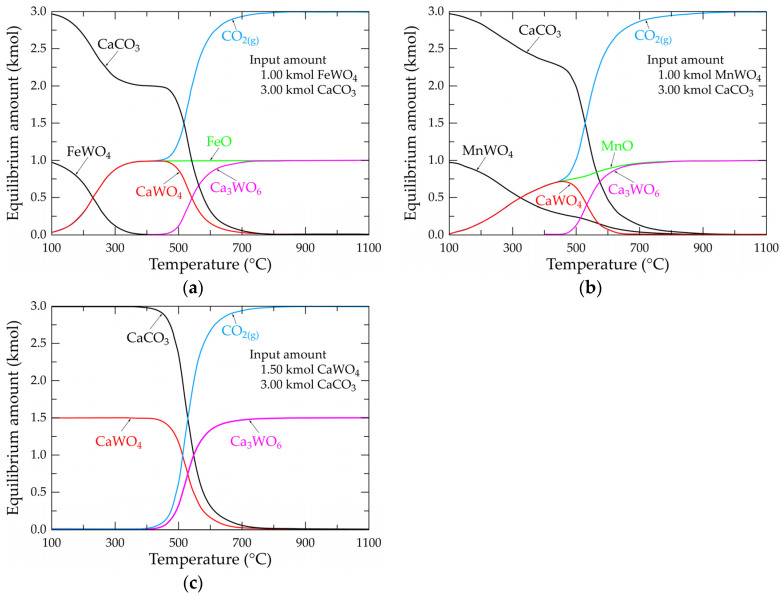
Evolution of the equilibrium composition as a function of temperature of the main selected species for systems (**a**) Fe-W-Ca-C-O; (**b**) Mn-W-Ca-C-O; (**c**) W-Ca-C-O.

Figure 7a shows the evolution of the molar composition of the Fe-Ca-W-C-O system by inputting 1.00 and 3.00 kmol FeWO_4_ and CaCO_3_, respectively, as starting compounds. To simplify the system, the calculation is made in the absence of oxygen. The species’ equilibrium composition evolves from 100 °C in the following two steps: the first step is up to 450 °C, with a rapid decrease in the amount of FeWO_4_ and CaCO_3_ and an increase in the amount of CaWO_4_, FeO and CO_2(g)_. Please note that the curves representing CaWO_4_, FeO and CO_2_ overlap up to 450 °C. This suggests that iron in the ferberite is substituted by calcium-generating scheelite. The second step (T > 450 °C) is characterized by the apparition of a new chemical species (Ca_3_WO_6_) reaching the value of 1.00 kmol at around 725 °C, while the amount of CaWO_4_ tends towards zero at about 725 °C. From this temperature, the equilibrium composition of the system consists of 1.00 kmol Ca_3_WO_6_, 1.00 kmol FeO and 3.00 kmol CO_2(g)_, which is consistent with the overall reaction described by Equation (3). Similar observations are valid for the Mn-Ca-W-C-O system (Figure 7b), but the equilibrium evolution is shifted towards higher temperatures. The final equilibrium composition at temperatures near 900 °C is consistent with the stoichiometry of the reaction given in Equation (9).

The simpler presentation is the W-Ca-C-O system, shown in Figure 7c. The evolution of the equilibrium composition starts at temperatures approaching 400 °C and ends at those near 800 °C, with an equilibrium composition of Ca_3_WO_6_ + CO_2(g)_ and with a molar ratio of Ca_3_WO_6_/CO_2(g)_ = 2, which is in agreement with the stoichiometry of the reaction given in Equation (15).

## 5. Calculation of the Equilibrium Composition in the Ca-W-Mg-O-Cl System

**Figure 8:**
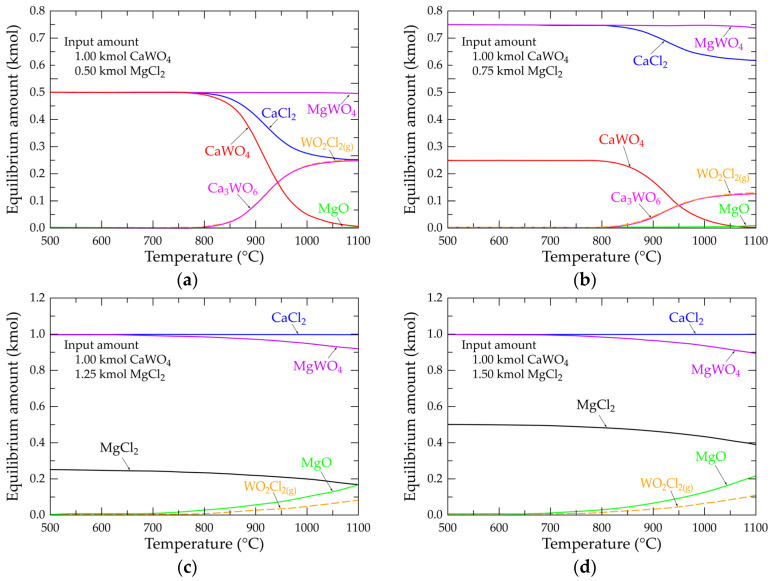
Evolution of the equilibrium composition as a function of temperature of the main selected species for systems Ca-W-Mg-O-Cl at (**a**) 0.50 kmol MgCl_2_; (**b**) 0.75 kmol MgCl_2_; (**c**) 1.25 kmol MgCl_2_; (**d**) 1.50 kmol MgCl_2_.

In the previous section, the equilibrium amount of main constituents in the Ca-W-Mg-O-Cl system was calculated at temperatures up to 1100 °C. Calculations are performed in the absence of oxygen atmosphere. Although the reaction of CaWO_4_ with MgCl_2_ (Equation (18)) requires an equimolar amount of CaWO_4_ and MgCl_2_, it was pertinent for modeling the equilibrium amount by varying the MgCl_2_ input from 0.50 to 1.50 kmol, while the amount of CaWO_4_ was kept constant and equal to 1.00 kmol. The obtained equilibrium diagrams are shown in Figure 8. Using 0.50 kmol MgCl_2_ in the starting materials (Figure 8a) and for temperatures lower than 800 °C, the equilibrium amount of the system is composed of equimolar quantities (0.50 kmol) of CaWO_4_, MgWO_4_ and CaCl_2_. Beyond 800 °C, two main species (Ca_3_WO_6_ and WO_2_Cl_2(g)_) appear and their amounts increase with the temperature. According to these observations, the likely reaction, thus producing these species, is that described by Equation (20), indicating that CaWO_4_ and CaCl_2_ are incompatible from a thermodynamic point of view. The calculation with 0.75 kmol MgCl_2_ (Figure 8b) also indicates the presence of these two species (Ca_3_WO_6_ and WO_2_Cl_2(g)_), but at lower relative amounts. At higher temperatures, magnesium oxide also belongs to the composition of the studied system. The equilibrium composition diagram with 1.00 kmol MgCl_2_ is predominated by an equimolar amount of MgWO_4_ and CaCl_2_ which is consistent with the stoichiometry of Equation (18).

Figure 8c,d shows the equilibrium compositions of the Ca-W-Mg-O-Cl system at 1.25 and 1.50 kmol MgCl_2_, respectively. The equilibrium quantities of CaCl_2_ remain constant (ca. 1.00 kmol) at all of the considered temperatures. However, the equilibrium amount of MgWO_4_ decreases with the temperature, and is more pronounced when 1.50 kmol MgCl_2_ is used in the system (Figure 8d). Additionally, equilibrium amounts of WO_2_Cl_2(g)_ and MgO increase significantly at temperatures above 800 °C. From these calculations and according to Equation (21), one may deduce that magnesium chloride can react with scheelite, thus generating calcium chloride and magnesium oxide. It is a spontaneous reaction, as is the case with a ΔG° value at 775 °C of −8.30 kJ/mol. The synthesis of tungsten oxychloride (WO_2_Cl_2(g)_) can also be described by Equation (22), but this reaction path seems improbable, as its ΔG° value at 775 °C is 72.17 kJ/mol.

The synthesis of metal oxychlorides, such as chromium oxychloride (CrO_2_Cl_2(g)_), during the oxychlorination of chromite (Fe,Mg)(Cr,Al,Fe)_2_O_4_ with a gaseous Cl_2_ + O_2_ mixture, is reported in previous works [20,21,22]; however, due to the high oxidation capacity of Cl_2(g)_ + O_2(g)_, chromium(III) is oxidized to chromium(VI), as seen in Equations (23) and (24), while in the case of scheelite, tungsten is already in a hexavalent state. Furthermore, the synthesis of WO_2_Cl_2(g)_, using CaWO_4_ and MgCl_2_ as starting materials, does not seem to be described in the literature.

2CaWO_4(s)_ + CaCl_2(s,l)_ → Ca_3_WO_6(s)_ + WO_2_Cl_2(g)_ (20)

1/2CaWO_4(s)_ + MgCl_2(s,l)_ → 1/2CaCl_2(s,l)_ + 1/2WO_2_Cl_2(g)_ + MgO_(s)_ (21)

MgWO_4(s)_ + MgCl_2(s,l)_ → WO_2_Cl_2(g)_ + 2MgO_(s)_ (22)

1/2FeCr^III^_2_O_4(s)_ + Cl_2(g)_ + 3/8O_2(g)_ → 1/4Fe_2_O_3(s)_ + Cr^VI^O_2_Cl_2(g)_ (23)

1/2MgCr^III^_2_O_4(s)_ + Cl_2(g)_ + 1/4O_2(g)_ → 1/2MgO_(s)_ + Cr^VI^O_2_Cl_2(g)_ (24)

## 6. Experimental Insights of Thermal Reactions of CaWO_4_ with CaCO_3_ and MgCl_2_

### 6.1. Materials and Expermental Procedure

**Figure 9:**
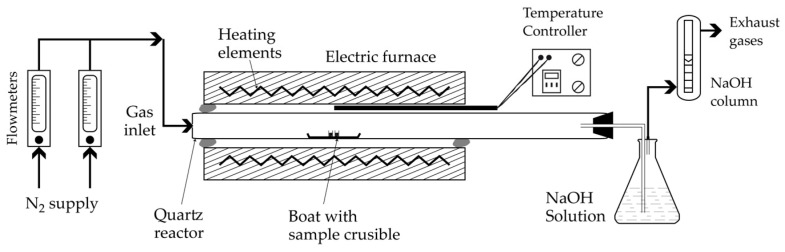
Schematic presentation of the experimental setup used for thermal tests.

Powdered analytical grade CaCO_3_, CaWO_4_ and MgCl_2_ samples are used is the study. Mixtures of CaWO_4_ + CaCO_3_ and CaWO_4_ + MgCl_2_ at defined proportions are produced by dry grinding. The elemental composition and crystallinity of the first two samples, as well as the solid reaction products, are determined using an energy dispersive spectroscopy electron microscopy (EDS-SEM, HITACHI S-4800, Hitachi Ltd., Tokyo, Japan) and X-ray diffraction (XRD, Bruker D8 Advance device, Bruker, Karlsruhe, Germany), respectively. No MgCl_2_ analysis was performed due to the highly hygroscopic nature of MgCl_2_. The experimental study results were achieved using the setup shown in Figure 9. The main piece of equipment is a tubular resistant furnace with the ability to reach 1500 °C. The working reactor is made of quartz capable of withstanding temperatures as high as 1300 °C in the absence of corrosive atmospheres. When the desired temperature is reached and stabilized under nitrogen flow, the alumina support containing the sample (approximately 1.8 g) is placed in the center of the furnace and the planned treatment time is allowed to elapse.

### 6.2. Reaction of CaWO_4_ with CaCO_3_

The formation of Ca_3_WO_6_ in the W-Ca-C-O system is demonstrated fairly well with thermodynamic assessments. To verify the reliability of thermodynamic calculation, a co-processing of pure CaWO_4_ and CaCO_3_ that have 15% more CaCO_3_ than the stoichiometric molar amount required by Equation (15) is performed in N_2_ gas flow at 850 °C, and the solid product obtained after a 120 min of treatment is examined with the XRD technique. Additionally, tests of thermal treatment of only CaCO_3_, in N_2_ and at various temperatures, show that at 850 °C for a duration time of 30 and 60 min, about 50% and 91% of CaCO_3_ are, respectively, decomposed into CaO, according Equation (25).

CaCO_3(s)_ → CaO _(s)_ + CO_2(g)_ (25)

**Figure 10:**
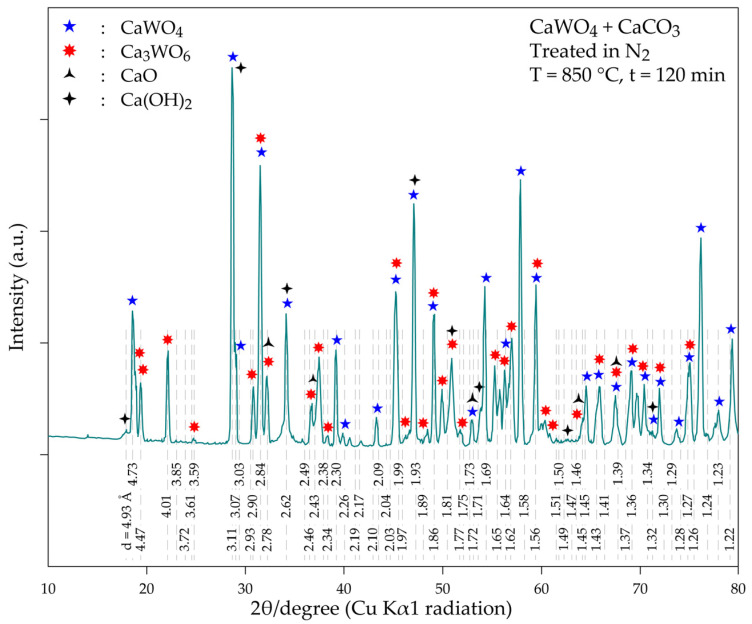
XRD diffractogram of the product obtained during the thermal treatment of a mixture of CaWO_4_ + CaCO_3_ at 850 °C for 120 min.

The analysis of the XRD diffractogram of the (CaWO_4_ + CaCO_3_) treatment product (Figure 10) confirms the presence of the well-crystallized Ca_3_WO_6_ with its main XRD peaks (d = 2.84 Å, 4.01 Å and 4.70 Å). However, the initial constituent (CaWO_4_) is still the predominant phase (d = 3.11 Å, 4.76 Å and 1.93 Å). In addition, CaO and Ca(OH)_2_ (the latter being the product of the interaction of CaO with moisture during sample preparation) are also identified in the thermal treatment sample. This finding indicates that although the reactions of CaCO_3_ (CaO) with CaWO_4_ are spontaneous processes (at least at T > 600 °C: Equations (13) and (15) and Figure 7c), temperatures higher than 850 °C and/or after an extended time of treatment should be applied for complete conversion of CaWO_3_ into Ca_3_WO_6_. One may conclude that the reactions’ kinetics are the limiting steps of the overall process. Further experimental tests have to be performed to obtain insight into the reaction mechanism and kinetics.

### 6.3. Reaction of CaWO_4_ with MgCl_2_

To validate the thermochemical calculation in the CaWO_4_-MgCl_2_ system, one of the preliminary thermal tests of the CaWO_4_ + MgCl_2_ mixture (MgCl_2_/CaWO_4_ = 1.25: molar ratio) is carried out at 775 °C under nitrogen atmosphere for 2 h. This temperature is chosen for at least three reasons, such as the following (i) to follow the thermodynamic predictions; (ii) to insure homogenous mixing between the CaWO_4_ solid and MgCl_2_ liquid (as the m.p. of MgCl_2_ is 714 °C [2]); (iii) to avoid the loss of MgCl_2(l)_ by evaporation, as this is expected at a temperature higher than 800 °C. The product of the thermal treatment is then washed with deionized water in order to eliminate the chlorides (MgCl_2_ and CaCl_2_), which cause problems for SEM-EDS and XRD sample preparation and analysis. Chlorides are hygroscopic (easily converted to (Mg,Ca)Cl_2_·6H_2_O, thus absorbing atmospheric moisture), making their handling and correct consistent analysis difficult. The solid mass after filtration and drying is examined using the XRD and SEM-EDS techniques.

**Figure 11:**
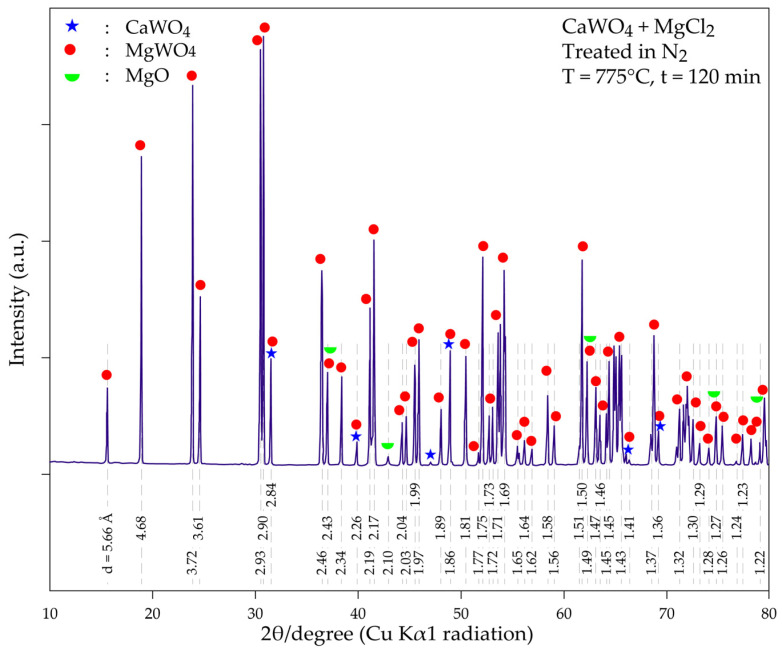
XRD diffractogram of the product obtained during the thermal treatment of a mixture of CaWO_4_ + MgCl_2_ at 775 °C for 120 min.

As shown in Figure 11, the magnesium tungstate phase (MgWO_4_) is well-crystallized with main peaks (d = 4.69 Å, 3.71 Å, 2.93 Å, 2.90 Å and 3.61 Å) and many other diffraction peaks, which predominate the obtained X-ray diffractogram. CaWO_4_ appears to be transformed into MgWO_4_. Although some diffraction peaks of CaWO_4_ is found overlapping with those of MgWO_4_, the presence of CaWO_4_ in the product is doubtful or occur as minor phases (i.e., if even present). Several characteristic X-ray peaks of magnesium oxide (MgO) are also revealed in the treatment product, although they are of low intensity. Its presence in the treatment residue may be explained by the reaction given above in Equation (21). Although pure nitrogen is used for experimental testing, traces of oxygen and/or moisture in the system can also result in the conversion of MgCl_2_ and MgO.

**Figure 12:**
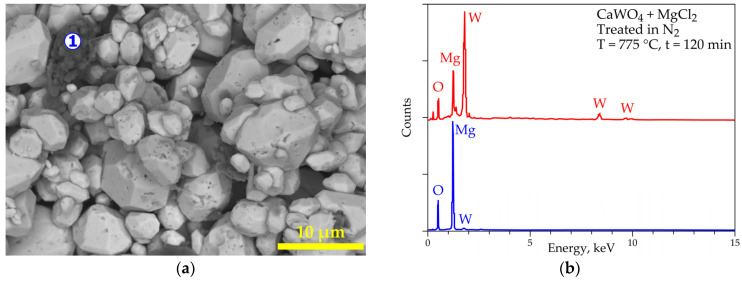
SEM-EDS results of the (CaWO_4_ + MgCl_2_) sample treated at 775 °C for 120 min in nitrogen atmosphere with spot “1” indicating EDS microanalysis. (**a**) General view (backscattered electron “BSE” micrograph) of the obtained sample. (**b**) EDS analysis of spot “1” (bleu spectrum); red spectrum represents overall EDS results of other homogenous particles.

SEM-EDS results of the treated product are reported in Figure 12a,b. Its morphology shows homogenous particles with sizes less than 10 µm (Figure 12a). Furthermore, another minor particle (spot n° 1) is observed. The general EDS spectrum (red spectrum in Figure 12b) clearly shows the presence of MgWO_4_, while the analysis of spot n°1 reveals the presence of MgO. These observations confirm the results obtained by XRD analyses; however, the presence of calcium (as CaWO_4_) is not revealed by SEM-EDS, which can be explained as either a complete transformation of CaWO_4_ into MgWO_4_ or the unreacted CaWO_4_ located in the core of the coarse particles, which is not accessible by the EDS probe.

To have an accurate idea of the kinetics of the CaWO_4_-MgCl_2_ interaction, a set of experimental tests from 725 °C to 775 °C and a 7.5 to 120 min residence time are carried out. As in the previous case, the composition of the obtained products is systematically monitored using the XRD and SEM-EDS techniques.

**Figure 13:**
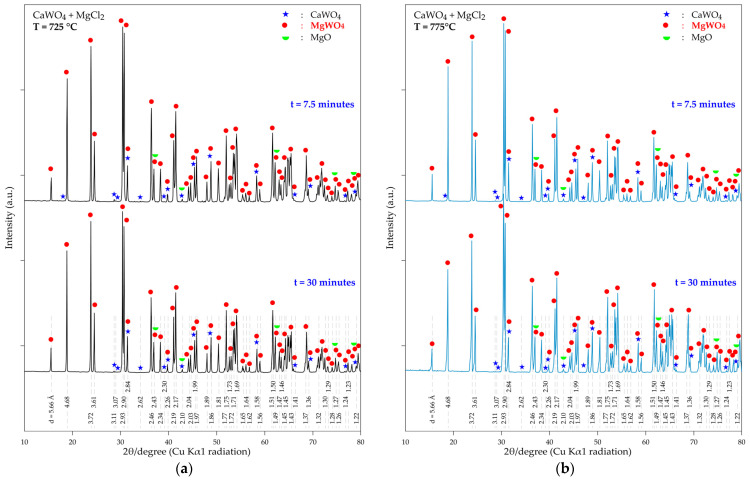
XRD patterns of the product obtained during the thermal treatment a mixture of CaWO_4_ + MgCl_2_ for 7.5 and 30 min at (**a**) 725 °C; (**b**) 775 °C.

The XRD patterns for a short-time treatment (7.5 and 30 min) of the resulting products are shown in Figure 13a,b. It is of note that, even at 725 °C, MgWO_4_ is the predominant phase when the sample is treated for 7.5 min. Additionally, typical peaks of CaWO_4_ are observed, although they are of low intensity. Similar observations are valid for the treatment at 775 °C (Figure 13b). This finding suggests that the kinetic reactivity of MgCl_2_-CaWO_4_ is high in the temperature range studied, confirming the thermodynamic predictions discussed earlier.

In a recent study [19], it is suggested that MgCl_2_ can be used for the extraction of tin from cassiterite (SnO_2_) materials via the formation of SnCl_4(g)_ at around 800 °C. Inferentially, magnesium chloride should also be used for the removal of tin from tungsten ores and concentrates containing SnO_2_ as an impurity prior to the hydrometallurgical extraction of tungsten.

## 7. Conclusions

This study provides some thermodynamic analysis and a modeling approach for the possible conversion of wolframite, and principally scheelite (CaWO_4_), into other compounds that are more suitable for subsequent hydrometallurgical processing.

Although treatment of the mineral with CaCO_3_ seems to be a feasible process thermodynamically, a satisfactory formation of the reaction products is not observed, which may be attributed to kinetic limitations. Thus, higher temperatures may be required for the treatment of scheelite with this chemical reagent.

Magnesium chloride, on the other hand, appears to be a useful chemical reagent for a satisfactory conversion of scheelite to magnesium tungstate (MgWO_4_). Equilibrium-phase diagrams suggest favorable conditions for the transformation of CaWO_4_ to MgWO_4_. Experimental results of the thermal treatment of scheelite are consistent with the thermodynamic predictions at moderate temperatures with acceptable kinetics. The study reveals that relatively lower temperatures (a little higher than 700 °C) are fine for this process; however, temperatures around 800 °C may be ideal due to the necessity involved in the removal of impurities such as tin (cassiterite), which may be associated with scheelite.

Future work will be devoted to thermochemical investigations on more complex systems, followed by extensive experimental tests on wolframite and scheelite concentrates and their mixtures, thus leading to the synthesis of easily leachable tungsten compounds under mild conditions.

## Data Availability

The original contributions presented in the study are included in the article, further inquiries can be directed to the corresponding authors.
